# Gilteritinib为基础的方案桥接异基因造血干细胞移植对复发难治伴FLT3-ITD突变急性髓系白血病患者的疗效及安全性研究

**DOI:** 10.3760/cma.j.cn121090-20231207-00297

**Published:** 2024-04

**Authors:** 洋 徐, 剑 张, 胜利 薛, 瞄 苗, 荧 王, 苏宁 陈, 惠英 仇, 德沛 吴

**Affiliations:** 苏州大学附属第一医院血液内科，江苏省血液研究所，国家血液系统疾病临床医学研究中心，苏州 215006 The First Affiliated Hospital of Soochow University, Jiangsu Institute of Hematology, National Clinical Research Center for Hematologic Diseases, Suzhou 215006, China

**Keywords:** Gilteritinib, 白血病，髓系，急性, 难治, 复发, FLT3-ITD基因突变, 异基因造血干细胞移植, Gilteritinib, Leukemia, myeloid, acute, Refractory, Relapse, FLT3-ITD mutation, Allogeneic hematopoietic stem cell transplantation

## Abstract

**目的:**

探讨Gilteritinib（Gilt）为基础的方案桥接异基因造血干细胞移植（allo-HSCT）对伴FMS样酪氨酸激酶3（FLT3）基因内部串联重复（ITD）突变的复发难治性急性髓系白血病（R/R AML）患者的安全性、有效性，以及移植后Gilt维持治疗对FLT3-ITD阳性R/R AML患者预后的影响。

**方法:**

回顾性分析2019年8月至2023年1月苏州大学附属第一医院收治的26例伴FLT3-ITD突变的R/R AML患者。统计所有纳入患者的复合完全缓解（CRc）率、总生存（OS）期、无病生存（DFS）期和不良反应。

**结果:**

26例FLT3-ITD突变阳性R/R AML患者中，男14例，女12例，中位年龄38（18～65）岁。难治18例，复发8例。用药第14～21天疗效：完全缓解（CR）率为26.9％（7/26），CR伴血液学不完全恢复（CRi）率为57.7％（15/26），部分缓解（PR）率为7.7％（2/26），CRc率为84.6％（22/26），微小残留病（MRD）转阴率为65.4％。所有患者12、24个月累计OS率分别为79.0％和72.0％。中位OS期未达到。中位随访时间为16.0个月。全部治疗有效患者12、24个月累计DFS率分别为78.0％和71.0％。中位DFS期未达到。接受allo-HSCT的患者中位OS期未达到，较未接受allo-HSCT的患者（3.3个月，95％ *CI* 2.2～4.3个月）显著延长（*P*＝0.005）。移植后是否应用Gilt维持治疗的患者中位OS期均未达到，且移植后维持治疗的患者OS明显优于移植后未进行维持治疗的患者（*P*＝0.019）。本研究中FLT3-ITD基因突变清除率为38.5％，且FLT3-ITD基因突变转阴的患者中位OS期未达到，明显长于未转阴的患者（15.0个月）（*P*＝0.018）。Gilt为基础的方案最常见的3级及以上血液学不良反应包括白细胞减少（76.9％）、中性粒细胞减少（76.9％）、中性粒细胞减少性发热（61.5％）、血小板减少（69.2％）和贫血（57.7％）。其中1例患者在移植后口服Gilt维持治疗时出现分化综合征，治疗后好转。

**结论:**

Gilt为基础的方案桥接allo-HSCT治疗FLT3-ITD突变阳性R/R AML患者的CRc率较高，MRD转阴率也较高，起效迅速，有效延长患者生存期。此外，FLT3-ITD基因突变清除率较高，且桥接移植和移植后Gilt维持治疗明显改善患者生存。治疗过程中不良事件的监测和管理至关重要。

## Abstract (en)

**Objective:**

This study aims to evaluate the safety and effectiveness of gilteritinib（Gilt）-based combination therapy bridging allo-HSCT for FLT3-ITD^+^ R/R AML. Additionally, it aims to assess the impact of Gilt maintenance therapy on the prognosis of patients after allo-HSCT.

**Methods:**

The clinical data of 26 patients with FLT3-ITD^+^ R/R AML treated at the First Affiliated Hospital of Soochow University from August 2019 to January 2023 were retrospectively analyzed. The analysis included an assessment of the composite complete remission rate（CRc）, overall survival（OS）time, disease-free survival（DFS）time, and adverse events experienced by all enrolled patients.

**Results:**

A total of 26 patients with FLT3-ITD^+^ R/R AML were enrolled, including 14 men and 12 women with a median age of 38（18–65）years. A total of 18 cases were refractory, and eight cases were relapsed. The curative effect evaluation conducted between 14 and 21 days showed that the complete remission（CR）rate was 26.9％（7/26）, the CR with hematology incomplete recovery was 57.7％（15/26）, and the partial response（PR）rate was 7.7％（2/26）. The CRc was 84.6％（22/26）, and the minimal residual disease（MRD）negativity rate was 65.4％. The 12 month cumulative OS rate for all patients was 79.0％, and the 24 month cumulative OS rate was 72.0％. The median OS time was not determined. The median follow-up time was 16.0 months. Among the patients who responded to treatment, the 12 month cumulative DFS rate was 78.0％, and the 24 month cumulative DFS rate was 71.0％. The median DFS time was not determined. Patients who received allo-HSCT had a median OS time that was significantly longer than those who did not receive allo-HSCT（3.3 months, 95％ *CI* 2.2–4.3 months, *P*＝0.005）. The median OS time of patients with or without Gilt maintenance therapy after allo-HSCT was not determined, but the OS time of patients with Gilt maintenance therapy after allo-HSCT treatment was longer than that of patients without Gilt maintenance therapy after allo-HSCT treatment（*P*＝0.019）. The FLT3-ITD mutation clearance rate in this study was 38.5％, and the median OS time of patients with FLT3-ITD mutation clearance was not determined but was significantly longer than the median OS of patients without FLT3-ITD mutation clearance（15.0 months; *P*＝0.018）. The most common grade 3 and above hematological adverse events of Gilt-based combination therapy included leukopenia（76.9％）, neutropenia（76.9％）, febrile neutropenia（61.5％）, thrombocytopenia（69.2％）, and anemia（57.7％）. One patient developed differentiation syndrome during oral Gilt maintenance therapy after allo-HSCT treatment, but his condition improved after treatment.

**Conclusion:**

The Gilt-based combination therapy is highly effective in treating FLT3-ITD^+^ R/R AML. It demonstrates a high CRc, MRD negativity rate, and rapid onset, leading to a significant improvement in patients' survival. Furthermore, the clearance rate of FLT3-ITD mutation is notably high. Additionally, implementing bridging allo-HSCT and Gilt maintenance therapy after allo-HSCT treatment has considerably enhances patients' survival. Closely monitoring and managing any adverse event that may occur during treatment are crucial.

FMS样酪氨酸激酶3（FLT3）基因突变是急性髓系白血病（AML）患者中最常见的遗传改变和不良预后因素，对AML患者的总生存（OS）期、无复发生存（RFS）期和无事件生存（EFS）期产生负面影响[1]–[2]。2022年欧洲白血病网（ELN）指南中指出，在风险分类中不再考虑FLT3-ITD等位基因比值，无论等位基因比值是高还是低或NPM1突变是否同时存在，所有伴有FLT3-ITD的AML（无不良风险遗传损伤）都被归类为中等风险组[3]。在2021年针对FLT3突变阳性AML的治疗指南中，建议在诊断时对FLT3基因突变进行快速分子检测，并应尽早考虑使用靶向药物以达到更深度缓解，同时应尽早考虑进行异基因造血干细胞移植（allo-HSCT）[4]。由于大剂量化疗和allo-HSCT不能充分改善其预后，FLT3激酶抑制剂的临床开发便为FLT3-ITD突变阳性复发难治（R/R）AML患者开辟了靶向治疗的前景，然而，关于Gilteritinib（Gilt）为基础的方案桥接allo-HSCT的疗效及安全性，以及移植后Gilt维持治疗对FLT3-ITD突变阳性R/R AML患者预后的影响，还需要进行更深入的研究和探索。本中心应用Gilt为基础的方案桥接allo-HSCT治疗26例FLT3-ITD突变阳性R/R AML患者，现将其疗效及安全性报道如下，为该方案在FLT3-ITD突变阳性R/R AML患者中的临床应用提供更多证据支持。

## 病例与方法

1. 病例：本研究回顾性分析2019年8月至2023年1月在苏州大学附属第一医院血液科住院治疗的26例伴FLT3-ITD突变的R/R AML患者的临床资料。诊断依据《中国复发难治性急性髓系白血病诊疗指南（2021年版）》[5]。所有患者根据骨髓细胞形态学、免疫表型分析、细胞遗传学、分子生物学进行诊断分型并确诊。FLT3-ITD等位基因比值≥0.5定义为高频。采用美国东部肿瘤协作组（ECOG）体能状态评分评估所有患者的日常活动能力。

2. 治疗方案：所有患者均签署了治疗知情同意书。治疗以Gilt为基础的方案桥接allo-HSCT及移植后Gilt维持。Gilt标准方案：120 mg/d，口服。药物用量及疗程根据不良反应及一般状况调整，出现粒细胞缺乏的患者，Gilt减量至80 mg/d。重度粒细胞减少（<0.5×10^9^/L）合并重度感染或出现严重不良反应时暂停用药。联合用药方案包括Gilt+维奈克拉（Ven）±阿扎胞苷（AZA）/地西他滨（DAC）（21例）、Gilt+Ven+DAC+克拉屈滨（Cladribine）（1例）、Gilt+IA（1例）和Gilt+AZA+半程HAAG方案（3例）。联合用药方案中Ven的用法：100 mg第1天，200 mg第2天，400 mg第3～14天，口服，出现中性粒细胞减少而接受伏立康唑/泊沙康唑预防真菌感染的患者，Ven剂量减至100 mg/d，出现严重粒细胞缺乏（<0.2×10^9^/L）并伴有严重的感染时停止口服Ven。其他药物的用法：Gilt+Ven±AZA/DAC方案：AZA 75 mg/m^2^第1～7天，静脉滴注；或DAC 20 mg/m^2^第1～5天，静脉滴注。Gilt+Ven+DAC+Cladribine：DAC 20 mg/m^2^第1～5天，静脉滴注；Cladribine 5 mg/m^2^，第1～5天，静脉滴注。Gilt+IA方案：去甲氧柔红霉素（IDA）12 mg/m^2^第1～3天，静脉滴注；阿糖胞苷（Ara-C）100～200 mg/m^2^，第1～7天，静脉滴注。Gilt+AZA+半程HAAG方案：AZA 75 mg/m^2^第1～7天，静脉滴注；高三尖杉酯碱1 mg/m^2^第3～7天，静脉滴注；Ara-C 10 mg/m^2^第3～7天，皮下注射；阿柔比星10 mg/m^2^第3～5天，静脉滴注；G-CSF根据血象调整，皮下注射。移植后患者Gilt 40～80 mg/d维持治疗（14例）。骨髓抑制期HGB<60 g/L或出现明显症状时输注悬浮红细胞，PLT<20×10^9^/L或出现出血倾向时输注血小板。治疗期间根据患者耐受情况及不良事件等对Gilt、Ven剂量进行调整，并适当停用化疗方案。化疗同时给予支持治疗，保护基本器官功能。伴细菌感染患者给予敏感抗生素治疗。老年或免疫缺陷患者给予伏立康唑/泊沙康唑等唑类药物预防真菌感染。

3. 疗效和不良反应：依据2022 ELN指南[3]进行疗效评价，包括完全缓解（CR）、CR伴血液学不完全恢复（CRi）、形态学无白血病状态（MLFS）、部分缓解（PR）、未缓解（NR）、复合完全缓解（CRc），其中CRc率为CR率+CRi率。原发难治性疾病：2个疗程强化诱导治疗后未见CR或CRi。血液系统复发：骨髓原始细胞≥5％，或者外周血中重现原始细胞，或发生髓外疾病[3]。OS期定义为开始应用Gilt至任何原因死亡或末次随访的时间。无病生存（DFS）期定义为治疗有效的患者，从评估治疗有效之日起到疾病复发、进展、任何原因死亡或末次随访的时间。依据NCI CTCAE 5.0进行不良反应评价[6]。微小残留病（MRD）的检测采用多参数流式细胞术（MCF）方法，MRD阴性缓解参照2022 ELN指南的MRD标准判定[3]。FLT3-ITD采用PCR毛细管电泳法验证，FLT3-ITD清除定义为FLT3-ITD变异等位基因频率≤10^−4^。

4. 随访：采用电话、查阅患者住院病历及门诊病历的方式随访，随访截止日期为2023年11月1日，中位随访时间为16.0（95％ *CI* 13.5～18.5）个月，所有患者均未失访。

5. 统计学处理：采用SPSS 26.0软件进行统计学分析，计数资料以例数表示；生存分析采用Kaplan-Meier法，差异性检验采用Log-rank法，*P*<0.05为差异有统计学意义。

## 结果

1. 一般资料：患者基本临床特征见表1。26例FLT3-ITD突变阳性R/R AML患者中，男14例，女12例，中位年龄38（18～65）岁。其中难治18例，复发8例，有4例患者在应用Gilt治疗后出现疾病复发。按照2022年ELN AML危险度分层体系，将患者分为预后中等组18例和预后不良组8例。初诊时中位骨髓原始细胞比例为74.5％（20.0％～96.5％）。仅有1例患者在接受Gilt治疗前接受过索拉非尼治疗。在本研究中，23例（88.5％）患者在接受Gilt治疗后桥接了allo-HSCT，14例（53.8％）患者在allo-HSCT后应用Gilt维持治疗，3例（11.5％）患者未桥接allo-HSCT。Gilt的中位治疗时间为6.5（0.6～13.6）个月。应用Gilt为基础联合治疗后达到首次完全缓解（CR_1_）的中位时间为22（14～36）d。本研究中，26例患者的基因突变涉及17种基因，中位伴随基因突变数为3（1～7）种，患者基因突变图谱见图1。FLT3-ITD突变同时伴有的突变率较高的前5种基因分别为WT1（34.6％）、DNMT3A（26.9％）、NPM1（26.9％）、RUNX1（23.1％）和TET2（15.4％）。患者开始应用Gilt时的ECOG评分：1分6例（23.1％），2分20例（76.9％）。

**表1:** 26例FLT3-ITD突变阳性复发难治急性髓系白血病患者基本临床资料

临床特征	数值
年龄[岁，*M*（范围）]	38（18~65）
性别[例（%）]	
男	14（53.8）
女	12（46.2）
初诊时骨髓原始细胞[%，*M*（范围）]	74.5（20.0~96.5）
诊断[例（%）]	
难治	18（69.2）
复发	8（30.8）
预后分层[例（%）]	
中等	18（69.2）
不良	8（30.8）
基因突变[例（%）]	
FLT3-ITD	26（100）
FLT3-ITD伴WT1	9（34.6）
FLT3-ITD伴DNMT3A	7（26.9）
FLT3-ITD伴NPM1	7（26.9）
FLT3-ITD伴RUNX1	6（23.1）
FLT3-ITD伴TET2	4（15.4）
用药方案[例（%）]	
Gilt+Ven±AZA/DAC	21（80.8）
Gilt+Ven+DAC+Cladribine	1（3.8）
Gilt+IA	1（3.8）
Gilt+AZA+半程HAAG	3（11.5）
疗效[例（%）]	
CR	7（26.9）
CRi	15（57.7）
PR	2（7.7）
NR	2（7.7）
桥接移植[例（%）]	
是	23（88.5）
否	3（11.5）
达到CR_1_的时间[d，*M*（范围）]	22（7~36）
脱离粒细胞缺乏的时间[d，*M*（范围）]	22.5（0~60）
其他FLT3抑制剂暴露史	
索拉非尼	1（3.8）
无	25（96.2）
开始用Gilt时ECOG评分[例（%）]	
1分	6（23.1）
2分	20（76.9）
应用Gilt时是否存在高频FLT3-ITD基因突变[例（%）]
是	16（61.5）
否	10（38.5）

**注** Gilt：Gilteritinib；Ven：维奈克拉；AZA：阿扎胞苷；DAC：地西他滨；Cladribine：克拉屈滨；IA：去甲氧柔红霉素、阿糖胞苷；HAAG：高三尖杉酯碱、阿糖胞苷、阿柔比星、G-CSF；CR：完全缓解；CRi：CR伴血液学不完全恢复；PR：部分缓解；NR：未缓解；CR_1_：首次完全缓解；ECOG：美国东部肿瘤协作组体能状态

**图1:**
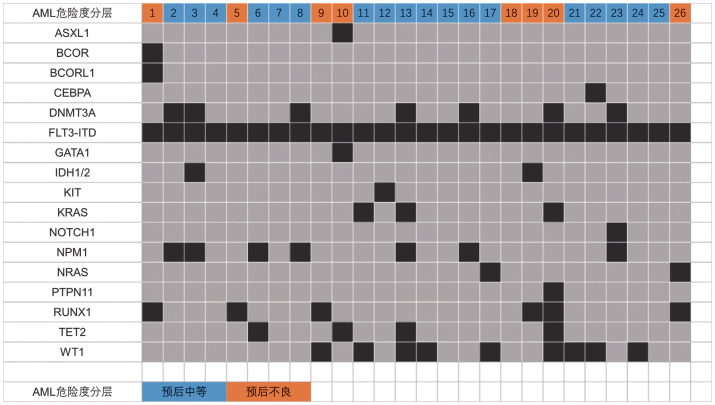
26例FLT3-ITD突变阳性复发难治急性髓系白血病（AML）患者基因突变图谱

2. 疗效评价：Gilt为基础联合治疗用药第14～21天疗效：CR率为26.9％（7/26），CRi率为57.7％（15/26），PR率为7.7％（2/26），CRc率为84.6％（22/26），MRD转阴率为65.4％（17/26），FLT3-ITD基因突变清除率为38.5％（10/26）。根据Gilt治疗反应分为有效组24例，无效组2例。根据是否桥接allo-HSCT将患者分为移植组和非移植组，移植组23例，有效21例，非移植组3例，全部有效。患者在应用Gilt时存在高频FLT3-ITD基因突变16例，全部有效，存在低频FLT3-ITD基因突变10例，有效8例。

所有患者12、24个月累计OS率分别为79.0％和72.0％。中位OS期未达到，平均OS期为23.4个月。中位随访时间为16.0（95％ *CI* 13.5～18.5）个月。全部治疗有效患者12、24个月累计DFS率分别为78.0％和71.0％。中位DFS期未达到。MRD转阴的患者与MRD未转阴的患者中位OS期均未达到，且差异无统计学意义（*P*＝0.200）。接受allo-HSCT的患者中位OS期未达到，与未接受allo-HSCT的患者（3.3个月，95％ *CI* 2.2～4.3个月）相比，差异有统计学意义（*P*＝0.005）（图2）。移植后是否应用Gilt维持治疗的患者中位OS期均未达到，且移植后维持治疗的患者OS期较未维持治疗的患者延长（*P*＝0.019）（图3）。应用Gilt时是否存在高频FLT3-ITD突变的患者中位OS期均未达到，且差异无统计学意义（*P*＝0.527）。FLT3-ITD突变转阴的患者中位OS期未达到，较FLT3-ITD突变未转阴的患者（15.0个月）延长（*P*＝0.018）（图4）。在WT1、DNMT3A、NPM1、RUNX1和TET2共突变基因亚组中，疗效和生存的分析比较均未显示出明显的统计学差异。

**图2:**
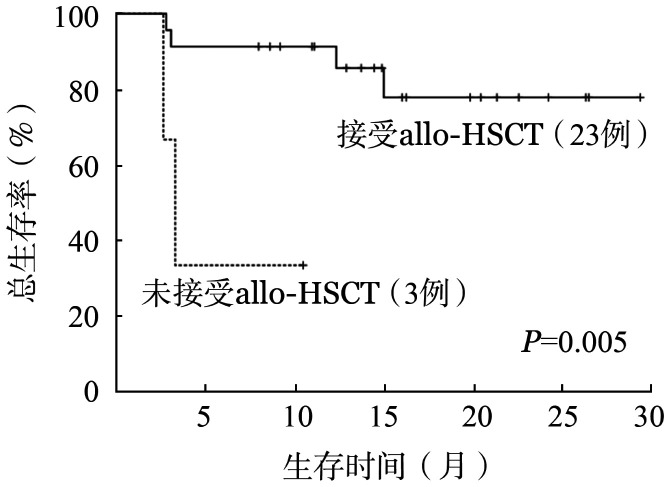
在Gilt为基础联合治疗后是否接受allo-HSCT对FLT3-ITD突变阳性复发难治急性髓系白血病患者总生存的影响 **注** allo-HSCT：异基因造血干细胞移植

**图3:**
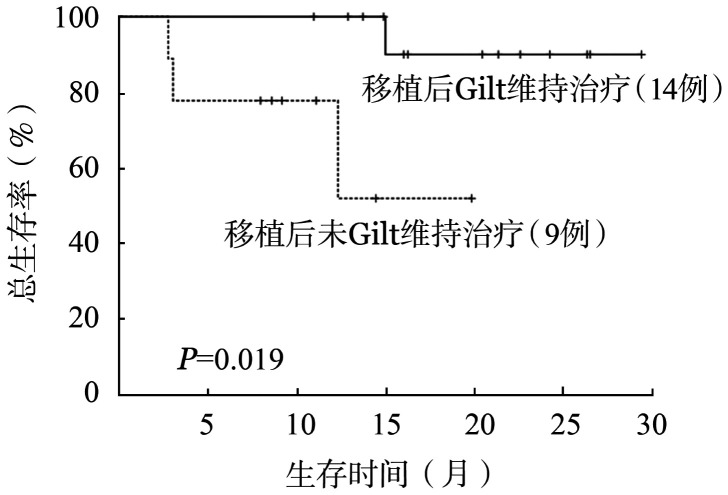
在进行移植后是否继续接受Gilt维持治疗对FLT3-ITD突变阳性复发难治急性髓系白血病患者总生存的影响

**图4:**
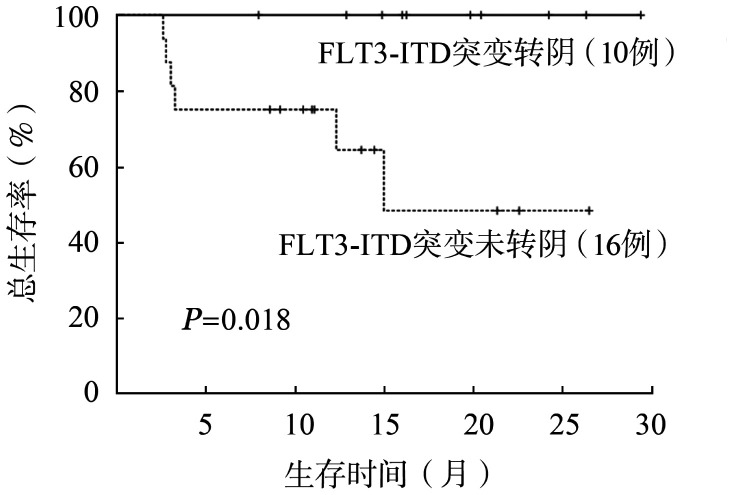
在Gilt为基础联合治疗后FLT3-ITD突变是否转阴对复发难治急性髓系白血病患者总生存的影响

在23例接受移植的患者中，14例接受亲缘单倍体造血干细胞移植，4例接受亲缘同胞全相合供者干细胞移植，5例接受无关全相合供者干细胞移植。其中20例使用改良BU-Cy方案预处理，1例使用Cy-TBI方案预处理，2例使用Flu-BU方案预处理。再缓解后到移植的中位时间为2.23个月，在此期间，7例患者在获得再缓解后直接桥接了allo-HSCT，14例患者以Gilt 120 mg为基础±Ven±AZA/DAC维持治疗，2例患者因未缓解，行挽救性allo-HSCT，并在该期间内为患者完善移植前准备。

3.不良反应：最常见的3级及以上血液学不良反应包括白细胞减少（20例，76.9％）、中性粒细胞减少（20例，76.9％）、中性粒细胞减少性发热（16例，61.5％）、血小板减少（18例，69.2％）和贫血（15例，57.7％）。其他常见不良反应为丙氨酸转氨酶（ALT）升高（10例，38.5％）、天冬氨酸转氨酶（AST）升高（9例，34.6％）。任何级别的胃肠道不良事件均常见，主要包括腹泻（6例，23.1％）、呕吐（5例，19.2％）和便秘（2例，7.7％）。26例患者中，8例因血细胞计数低而将Gilt剂量减至40～80 mg。其中5例为重度粒细胞减少（<0.5×10^9^/L）合并严重感染，停止口服Gilt。其中1例患者在移植后口服Gilt维持治疗时出现分化综合征，具体表现为发热、心包积液，停止口服Gilt，治疗后好转。骨髓抑制期监测血常规，HGB<60 g/L或出现明显贫血症状时输注悬浮红细胞。PLT<20×10^9^/L或有明显出血倾向时输注血小板。在口服给药第14～21天，进行骨髓细胞形态学检查。若原始细胞比例<5％，则加入G-CSF，直至中性粒细胞绝对计数（ANC）>0.5×10^9^/L，且脱离血小板输注，Gilt剂量增至120 mg/d。

接受allo-HSCT的23例患者中，6例（26.1％）发生移植物抗宿主病（GVHD），其中3例为0～Ⅰ度急性GVHD（aGVHD），3例为Ⅱ度aGVHD。发生皮肤排斥反应3例（Ⅰ级3例），肠道排斥反应1例（Ⅱ级），肺部排斥反应1例（表现为间质性肺炎），口腔排斥反应1例（表现为口腔黏膜炎），肝脏排斥反应1例（Ⅰ级）。3例皮肤排斥反应和1例肝脏排斥反应进展为慢性GVHD。

## 讨论

FLT3基因突变对预后的影响并不局限于新诊断的AML，在R/R AML患者中，伴FLT3基因突变预示着更差的预后和生存[7]。对于伴FLT3基因突变的患者，移植后2年的累计复发率可高达（30±5）％，且移植后2年内无白血病生存（LFS）率仅为（58±5）％[8]。Gilt作为新型的Ⅰ型抑制剂，是一种高选择性、强效的FLT3抑制剂，也是FLT3/ AXL的小分子双重抑制剂[9]。其于2018年被美国FDA批准用于治疗伴有FLT3基因突变的R/R AML，在NCCN、ESMO指南中，Gilt被优先推荐用于治疗FLT3突变阳性R/R AML[10]–[11]。该药物于2021年在我国上市，成为国内首个具有AML适应证的FLT3抑制剂，同时也为改善R/R AML患者的预后带来了希望。

其他关于应用FLT3抑制剂治疗R/R AML的临床试验仍在进行中。ADMIRAL临床试验中，对接受口服Gilt与接受挽救性化疗的R/R AML患者进行随访，Gilt组和挽救化疗组总反应率分别为67.6％和25.8％，Gilt组中位OS期长于挽救化疗组，且死亡风险降低33.5％[12]–[13]。本研究中，Gilt为基础联合治疗方案在FLT3-ITD突变阳性R/R AML患者中的CRc率为84.6％，MRD转阴率为65.4％。这一CRc率明显优于既往回顾性研究，其中，FLT3抑制剂暴露再接受以Ven为基础治疗方案的患者总反应率为56.8％[14]。应用Gilt后达到CR_1_的中位时间为22（14～36）d。中位随访时间为16.0个月，中位OS期未达到，平均OS期为23.4个月。本研究应用Gilt为基础的联合方案治疗FLT3-ITD突变阳性R/R AML患者，治疗CRc率较高，MRD转阴率也较高，起效迅速，有效延长患者生存期，显著改善这类患者的预后。

本研究中两例NR的患者，一例患者确诊AML-M_5_合并WT1突变，既往研究提示，FLT3激活在AML-M_5_患者中显著增加，且FLT3-ITD突变的存在与较差的临床反应相关[15]。与此同时，WT1突变作为单一分子标志物对预后没有影响，但WT1突变合并FLT3-ITD突变对预后具有不良的影响[16]。另一例患者确诊AML-M_4_合并DNMT3A、RUNX1、TET2、KRAS、WT1、PTPN11基因突变，按照ELN2022风险分类归为预后不良组，一项Gilt治疗FLT3突变的R/R AML患者的分子特征研究提示，对Gilt耐药的患者常发生的突变类型为Ras/MAPK通路基因突变以及FLT3 F691L基因突变，其中最常见的Ras/MAPK通路基因包括NRAS、PTPN11和KRAS[17]。Ras/MAPK通路基因突变与Gilt的耐药性相关[18]。

本研究将患者分为接受移植和未接受移植组，分析两组的OS，接受allo-HSCT的患者中位OS期未达到，与未接受allo-HSCT的患者（3.3个月）相比，差异有统计学意义（*P*＝0.005）。与一项Ⅰb期研究一致，Gilt联合Ven治疗FLT3突变的R/R AML患者，桥接allo-HSCT可获得更多生存获益[19]。

在ADMIRAL研究中，Gilt组接受移植的FLT3突变R/R AML患者，与移植后未重启Gilt维持治疗相比，重启Gilt维持治疗的患者有更长的OS期，更低的复发率[20]。本研究中，移植后是否应用Gilt维持治疗的患者中位OS期均未达到，且移植后维持治疗的患者OS期较移植后未进行维持治疗的患者长（*P*＝0.019）。这初步表明了移植后应用Gilt维持治疗可以显著改善患者的长期生存，与ADMIRAL研究一致，但确切的结论需要等待临床试验的结果。本研究中FLT3-ITD基因突变清除率为38.5％，且FLT3-ITD基因突变转阴的患者中位OS期未达到，与未转阴的患者（15.0个月）相比，差异有统计学意义（*P*＝0.018）。表明Gilt为基础联合治疗可使FLT3-ITD阳性R/R AML患者获得较高的FLT3-ITD基因突变清除率，且明显改善了FLT3-ITD转阴患者的长期生存和预后。

在本研究中，我们发现3级或更高级别的血液学不良反应（如白细胞减少、中性粒细胞减少、中性粒细胞减少性发热、贫血和血小板减少）以及其他常见不良反应（ALT、AST升高）和任何级别的胃肠道不良事件都较为常见。1例患者出现分化综合征，该患者停药并接受激素等治疗后好转。这些不良反应都在可控范围内，与既往研究一致，Gilt不论是作为单药治疗还是联合治疗方案的一部分，在治疗AML患者时都表现出良好的安全性[12]，[19]。

综上所述，对于伴有FLT3-ITD基因突变R/R AML患者，应用Gilt为基础联合治疗的CRc率较高，MRD转阴率也较高，起效迅速，生存期延长。此外，FLT3-ITD基因突变清除率较高，桥接移植和移植后Gilt维持治疗可明显改善患者生存和预后。尽管治疗过程中出现的不良反应均在可耐受范围内，但对不良事件的监测和管理仍至关重要。本研究为回顾性研究，入组患者例数偏少，尚需等待进一步的临床试验数据来支持本研究结果。
